# Extent of neck dissection for patients with clinical N1 oral cancer

**DOI:** 10.1007/s10147-020-01635-8

**Published:** 2020-03-05

**Authors:** Yasumasa Kakei, Hirokazu Komatsu, Tsutomu Minamikawa, Takumi Hasegawa, Masanori Teshima, Hirotaka Shinomiya, Naoki Otsuki, Ken-ichi Nibu, Masaya Akashi

**Affiliations:** 1 Department of Oral and Maxillofacial Surgery, Kobe University Graduate School of Medicine, Kobe, Japan; 2 Department of Otolaryngology, Kobe University Graduate School of Medicine, Kobe, Japan

**Keywords:** Tongue cancer, Clinical N1, Neck dissection, Level IV, Level V, Supraomohyoid neck dissection, N1, Oral cancer

## Abstract

**Background:**

No clear consensus has been reached on the indication of supraomohyoid neck dissection (SOHND) for clinically positive lymph-node metastasis.

**Patients:**

Consecutive 100 patients with previously untreated oral cancer treated at Kobe University Hospital were included in this study. All patients were clinically staged as anyTN1M0 and underwent radical dissection of the primary site and level I–V neck dissection as the initial treatment.

**Results:**

None of the 100 patients had pathological lymph-node metastasis (pLN) to level V. pLN to level IV was observed in two patients with tongue cancer in whom clinical lymph-node metastasis was preoperatively observed at level II.

**Conclusions:**

Level V may be excluded in the neck dissection for patients with N1 oral cancers. Level IV dissection should be considered in the patient with tongue cancer and clinical lymph-node metastasis at level II.

## Introduction

Supraomohyoid neck dissection (SOHND) is classified as a selective dissection of levels I, II, and III of the neck [1]. Since skip metastasis to level IV is rare in oral cancer [2] and recent advances in diagnostic imaging including enhanced computed tomography (CT), magnetic resonance imaging (MRI), as well as positron emission tomography/computed tomography (PET-CT) have provided precise preoperative evaluation of neck lymph-node metastases, a prospective trial on prophylactic neck dissections in oral cancer was conducted and found no significant difference in survival between modified radical neck dissection (MRND) and SOHND groups [3]. Now, SOHND has been accepted worldwide as a technique of prophylactic neck dissection for high-risk clinical N0 (cN0) tongue/oral cancers [4, 5]. Furthermore, sentinel node biopsy (SNB) has been gradually accepted as a reliable staging test for patients with early disease and radiologically N0 neck. Since SNB can detect occult metastases with a sensitivity of 86–94%, patients with no sign of metastases on SNB could avoid neck dissection [6].

On the other hand, no clear consensus has been reached on the clinical N1 (cN1) cases, although several studies recommended SOHND even for clinical N1 (cN1) cases in recent reports [7, 8]. To explore the optimal surgical procedure for cN1 oral cancers, in this study, we investigated the appropriateness of SOHND in patients with cN1 oral cancer through a retrospective review of pathological neck lymph-node metastases (pLN) among the patients who underwent level I–V neck dissection for the treatment of cN1 oral cancer at Kobe University Hospital.

## Materials and methods

This retrospective study included consecutive 100 patients with previously untreated oral squamous cell carcinoma, who were treated at Department of Oral and Maxillofacial Surgery or Department of Otolaryngology-Head and Neck Surgery, Kobe University Hospital, between January 1999 and December 2016. All the patients were preoperatively evaluated by CT and MRI. PET-CT was also employed in most cases. Ultrasound (US)-guided fine needle aspiration biopsy (FNAB) was performed as an additional examination when judging was difficult by CT, MRI, and PET-CT. Diagnostic criteria used for CT, MRI, PET-CT, and US in this study were described elsewhere [9, 10]. Cancers were clinically staged as anyTN1M0 according to the seventh edition of TNM Classification of Malignant Tumours (UICC), and underwent radical resection of the primary site and level I–V neck dissection as the first-line treatment. Patients underwent postoperative radiotherapy with or without cisplatin when patients were at high risks such as positive or close surgical margins, extranodal extension, or multiple lymph-node metastases. Patients were followed up at our outpatient clinic, monthly in the first year, bimonthly in the second year, and then trimonthly. In general, patients were evaluated by CT every 6 months and by PET-CT every year.

The patients’ ages ranged from 31 to 88 years (average, 68 years), with 58 males and 42 females. Most common primary site was tongue (45 patients), followed by lower gingiva (24), buccal mucosal (15), oral floor (8), and upper gingiva (8). As for T classification, 4 patients were classified as T1, 49 as T2, 22 as T3, and 25 as T4. Clinical and pathological data were obtained from medical records of the patients. Clinical distribution and pathological distribution of the lymph-node metastasis were evaluated according to the characteristics of the patients. This study was approved by the ethical committee of Kobe University Hospital (#1401) and written informed consent was obtained from all the patients.

## Results

### Preoperative level of clinical lymph-node metastasis

The preoperative levels of clinical lymph-node metastasis (cLN) are summarized in Table 1. Before surgery, cLN was observed at level I or II in all patients. No patients had cLN at level III, IV, or V. Among the patients with tongue cancer, metastasis to level II was common, observed in 25 (56%) of the 45 patients. Among the patients with buccal mucosal, oral floor, or gingival cancer, cLN was observed at level IB in 43 (78%) of the 55 patients, and only 12 (22%) had cLN at level II.

**Table 1:** Level of clinical lymph-node metastasis by primary subsite

	No. of pts	IA	IB	II	III	IV	V
Tongue	45	2	18	25	0	0	0
Lower gingiva	24	0	20	4	0	0	0
Buccal mucosa	15	0	12	3	0	0	0
Oral floor	8	0	5	3	0	0	0
Upper gingiva	8	0	6	2	0	0	0
Total	100	2	61	37	0	0	0

*No* number, *Pts* patients, *IA–V* levels IA–V of the neck

### Pathological lymph-node metastases and subsite of metastasis

At least one pLN was observed in 66 of the 100 patients. Thirty-seven patients had one pN (pLN1), and 29 had multiple pLNs (pN2b). The levels of the pLN farthest from the primary subsite (most distal metastasis) in individual patients are summarized in Table 2. In most patients, the most distal metastasis was at level III or nearer. Only two patients with pN2b had pLN at level IV, and both of them also had metastatic lymph nodes at level III. Metastasis to level V was not observed in any patient of this study.

**Table 2:** Distribution of pathological lymph-node metastases

	No. of pts	IA	IB	II	III	IV	V
pN1	37	2	20	14	1	0	0
pN2b	29	1	8	10	8	2	0
Total	66	3	28	24	9	2	0

*No* number, *Pts* patients, *IA–V* levels IA–V of the neck, *pN1* one pathological lymph-node metastasis, *pN2b* two or more pathological lymph-node metastases

*Based on the most distal level where lymph-node metastasis was pathologically identified in individual patients

### Level of pathological lymph-node metastasis according to the primary subsite

For the 66 patients who had pLN(s), the most distal levels of pLN were summarized by primary subsite in Table 3. Metastasis to level IV was observed only in two patients with tongue cancer, while the pLN of all other cancers were limited to level I–III. There was no significant difference in the number of pLNs between subsites or T stages (Table 4).

**Table 3:** Distribution of pathological lymph-node metastases by primary subsite

	pN+	pN1	pN2b	IA	IB	II	III	IV	V
Tongue	32/45 (71%)	18	14	1	10	14	5	2	0
Lower gingiva	11/24 (46%)	4	7	0	7	4	0	0	0
Buccal mucosa	11/15 (73%)	7	4	2	6	3	0	0	0
Oral floor	5/8 (63%)	3	2	0	1	1	3	0	0
Upper gingiva	7/8 (88%)	5	2	0	4	2	1	0	0
Total	66/100 (66%)	37	29	3	28	24	9	2	0

*pN+* pathological lymph-node positive, *pN1* one pathological lymph-node metastasis, *pN2* two pathological lymph-node metastases, *IA–V* levels IA to V of the neck

*Based on the most distal level where a lymph-node metastasis was pathologically identified in individual patients

**Table 4:** Distribution of pathological lymph-node metastases by clinical t stage

	pN+	pN1	pN2b	IA	IB	II	III	IV	V
T1	4/4 (100%)	4	0	0	3	1	0	0	0
T2	39/49 (80%)	17	19	2	15	11	6	2	0
T3	14/22 (64%)	7	7	0	4	8	2	0	0
T4	11/25 (44%)	9	3	1	6	4	1	0	0
Total	66/100 (66%)	37	29	3	28	24	9	2	0

*pN+* pathological lymph-node positive, *pN1* one pathological lymph-node metastasis, *pN2* two pathological lymph-node metastases, *IA–V* levels IA to V of the neck

*Based on the most distal level where a lymph-node metastasis was pathologically identified in individual patients

### Levels of clinical and pathological lymph-mode metastases

Among the patients with cLN in level I, the pLN(s) was limited to level I or II in 38 (90%) of the 42 subjects. Only four patients (10%) had metastasis at level III, and none at level IV. As for the patients with cLN to level II, five (21%) and two (0.8%) had pLN at level III and level IV, respectively (Table 5).

**Table 5:** Distribution of pathological lymph-node metastases by the level of clinical lymph-node metastasis

Clinical LN metastasis	Pathological LN metastasis						
	Total	IA	IB	II	III	IV	V
Level I	42/63	3	25	10	4	0	0
Level II	24/37	0	3	14	5	2	0
Total	66/100	3	28	24	9	2	0

*LN* lymph node, *IA–V* levels IA to V of the neck

*Based on the most distal level where a lymph-node metastasis was pathologically identified in individual patients

## Discussion

Andersen et al. performed selective neck dissection for 106 oral cancers with cLN(s) including 58 patients with cN1. With the positive results of a 5-year disease-specific survival of 88.1%, a 5-year failure rate in the neck of 6.7%, and a low regional recurrence rate of 4.3%, they concluded that selective neck dissection is useful even in patients with cLN(s), depending on the size and mobility of the lymph nodes and histories of prior neck surgery and/or radiotherapy [11]. Kowalski et al. [7] and Asakage et al. [8] have also acknowledged SOHND as an appropriate treatment option for N1 patients whose metastasis is limited to level I because of the low frequencies of metastasis to level IV between 0 and 0.6%. However, Shah et al. have reported that 15–16% of tongue/oral cancer with cLN(s) had pLN(s) to level IV [2], and many experts take a cautious stance on limiting (sparing) the extent of dissection. Suzuki et al. [12] recommended extended SOHND, which includes dissecting level IV, since 1 of the 52 patients with cN1 tongue cancer had pLN at level IV. Harada et al. examined 95 patients with oral cancer, with only one of them, a patient with tongue cancer, experiencing metastasis to level IV. As in our present study, oral cancer originating in other subsites did not spread to level IV [13]. These results suggest that in the treatment of anyTN1 oral cancer except of tongue, level IV dissection can be excluded, whereas when treating tongue cancer, we should be aware that metastasis to level IV can occur, although rare in frequency.

In the present study, two patients with tongue cancer who had pLN in level IV were preoperatively diagnosed as having cLN at level II. Extended SOHND including level IV should be considered, at least in the treatment of tongue cancer with cLN at level II. Shimbashi et al. have reported that 2 out of 22 patients with tongue cancer had pLN to level IV and, similar to our present results, both had multiple pLNs [14]. Accordingly, in our study, metastasis to level IV was accompanied by pLN in level III in both patients, without the so-called skip metastasis [15]. As proposed by Koerdt et al. [16], a strategy may be successful to consider dissecting level IV if metastatic lymph node at level II or III is identified by intraoperative frozen section.

Similar to reports from Japan and overseas [8, 12], pLN to level V was not observed among our patients either. Recent meta-analysis by Liang et al. found no significant difference between selective and radical neck dissection in terms of regional control and prognosis [17], suggesting a little impact of level V dissection on the prognosis of the patients with oral cancer. Moreover, avoiding the dissection of level V reduces postoperative damage to the accessory nerve [19]. Taken together, standard or extended SOHND excluding level V would be acceptable in the treatment of patients with cN1 oral cancers.

It should be noted that false-positive and false-negative rates of preoperatively diagnosed clinical lymph-node metastasis were relatively higher in the previous studies [5, 7]. Given the recent advances in the accuracy of diagnostic modalities such as US-guided FNAB and SNB, further research needs to be done in more strictly defined N1 (level I) patients. From the oncological point of view, survival rate of the patients with cN1/pN2b was unfavorable in the present study. Eight out of 29 patients with pN2b died of disease during the observation period. In the patients with clinically N1 and multiple occult histopathological metastases, the tumor may be high biological malignancy. Postoperative treatments, such as chemotherapy, radiotherapy, and/or immunotherapy, should be reconsidered, to control the primary tumor as well as regional and distant metastases for these patients.

Another limitation of this study is the TNM staging system employed in the analysis. Since we could not obtain the precise value of depth of invasion (DOI) of early tongue cancers in old cases, we staged according to the seventh TNM staging system, instead of eighth. Thus, we did not evaluate the correlation between DOI and metastasis to level III or IV in this analysis. Although there were no significant differences in the frequency of metastasis to level III or level IV according to T classification in the present study, multi-institutional prospective study using eighth TNM staging system [20] should be performed to draw more definitive conclusions.

Avoiding level IV dissection has several potential merits to prevent additional operation time and/or blood loss, chylorrhea, and phrenic nerve paralysis when less-experienced surgeons perform dissection of level IV. However, these merits are small for well-experienced surgeons. In addition, our previous studies have found no significant difference between levels I–III and I–IV dissections in terms of postoperative quality of life (QOL) and shoulder function, provided that neck rehabilitation was performed after surgery [19]. Besides the extent of dissection, providing effective postoperative rehabilitation is also an important issue.

## Conclusions

In conclusion, none of the 100 patients diagnosed as cN1 oral cancer had pLN to level V, suggesting that level V dissection can be excluded. Metastasis to level IV was observed only in two patients with tongue cancer who had a suspected metastasis at level II before surgery, both being pN2b subjects. SOHND is not necessarily an appropriate option for a cN1 patient with oral cancer when the cancer originates in the tongue, the patient has a suspicious lymph node at level II before surgery, and/or intraoperative diagnosis finds multiple lymph-node metastases. Further investigation with more patients is needed to find the range of patients for whom SOHND is indicated.
